# Dl-3-n-Butylphthalide Promotes Remyelination and Suppresses Inflammation by Regulating AMPK/SIRT1 and STAT3/NF-κB Signaling in Chronic Cerebral Hypoperfusion

**DOI:** 10.3389/fnagi.2020.00137

**Published:** 2020-06-09

**Authors:** Meixi Li, Nan Meng, Xin Guo, Xiaoli Niu, Zhongmin Zhao, Wei Wang, Xiaohua Xie, Peiyuan Lv

**Affiliations:** ^1^Department of Neurology, Hebei Medical University, Shijiazhuang, China; ^2^Department of Neurology, Hebei General Hospital, Shijiazhuang, China

**Keywords:** chronic cerebral hypoperfusion, Dl-3-n-butylphthalide (NBP), remyelination, inflammation, AMPK/SIRT1 pathway, STAT3/NF-κB pathway

## Abstract

Demyelination in vascular dementia (VD) is partly attributable to inflammation induced by chronic cerebral hypoperfusion (CCH). Remyelination contributes to the recovery of cognitive impairment by inducing the proliferation and differentiation of oligodendrocyte progenitor cells. It was previously reported that Dl-3-n-butylphthalide (NBP) promotes cognitive improvement. However, whether NBP can stimulate remyelination and suppress inflammation after CCH remains unclear. To answer this question, the present study investigated the effects of NBP on remyelination in a rat model of CCH established by bilateral carotid artery occlusion. Functional recovery was evaluated with the Morris water maze (MWM) test, and myelin integrity, regeneration of mature oligodendrocytes, and inhibition of astrocyte proliferation were assessed by immunohistochemistry and histologic analysis. Additionally, activation of 5′ AMP-activated protein kinase (AMPK)/Sirtuin (SIRT)1 and Signal transducer and activator of transcription (STAT)3/nuclear factor (NF)-κB signaling pathways was evaluated by western blotting. The results showed that NBP treatment improved memory and learning performance in CCH rats, which was accompanied by increased myelin integrity and oligodendrocyte regeneration, and reduced astrocyte proliferation and inflammation. Additionally, NBP induced the activation of AMPK/SIRT1 signaling while inhibiting the STAT3/NF-κB pathway. These results indicate that NBP alleviates cognitive impairment following CCH by promoting remyelination and suppressing inflammation *via* modulation of AMPK/SIRT1 and STAT3/NF-κB signaling.

## Introduction

Vascular dementia (VD), which accounts for around 15% of dementia cases, is a major threat to the health and productivity of the global population (O’Brien and Thomas, 2015) as it is associated with high morbidity and reduced cognitive function. White matter lesions (WMLs) and neuroinflammation are pathologic features of VD (Saggu et al., 2016; Hase et al., 2018). Moreover, the latter accelerates the apoptosis of oligodendrocytes, leading to demyelination and WMLs (Gouw et al., 2011). Myelin repair is critical for cognitive recovery (Li et al., 2017). Remyelination—which is often defective in demyelinating diseases including VD (Fancy et al., 2009; Jiang et al., 2017)—depends on the proliferation and differentiation of oligodendrocyte progenitor cells (OPCs; Franklin and Ffrench-Constant, 2008). Thus, therapeutic strategies that suppress inflammation and stimulate OPC differentiation into oligodendrocytes can alleviate cognitive impairment associated with VD by stimulating remyelination (Jiang et al., 2017; Figlia et al., 2018).

5′AMP-activated protein kinase (AMPK) is an evolutionarily conserved metabolic sensor that maintains energy balance at the cellular and systemic levels (Dasgupta and Milbrandt, 2009). Sirtuin (SIRT)1 is a deacetylase involved in gene expression, metabolism, and aging (Bonda et al., 2011; Paraiso et al., 2013). AMPK and SIRT1 engage in a mutual positive regulatory interaction (Ruderman et al., 2010) and prevent inflammation and demyelination in both physiologic and pathologic contexts (Giri et al., 2008; Elbaz et al., 2018; Dembic et al., 2019). However, little is known about the role of AMPK/SIRT1 signaling in VD-associated inflammation and demyelination.

Nuclear factor (NF)-κB is a transcription factor that has been implicated in the white matter changes observed in chronic cerebral hypoperfusion (CCH) rats (Saggu et al., 2016). Signal transducer and activator of transcription (STAT)3 functions as a pro-inflammatory cytokine in VD pathology (Won et al., 2013), and induces the differentiation of astrocytes and blocks that of oligodendrocytes from neural stem cells or OPCs (Sun et al., 2015; Imamura et al., 2017). STAT3 and NF-κB cross regulate and STAT3-induced NF-κB activation was shown to accelerate pathogenesis in neurodegenerative diseases (Grivennikov and Karin, 2010). SIRT1 was found to suppress STAT3/NF-κB signaling in inflammatory disorders (Kauppinen et al., 2013; Elbaz et al., 2018). However, it is unclear whether there is crosstalk between AMPK/SIRT1 and STAT3/NF-κB signaling in VD.

Dl-3-n-butylphthalide (NBP), a compound extracted from the seeds of Chinese celery, has been shown to alleviate cognitive impairment in VD models by inhibiting the apoptosis of hippocampal neurons (Xu et al., 2017; Li et al., 2019a), endoplasmic reticulum stress (Niu et al., 2019), and oxidative stress (Qi et al., 2018), and improving hemodynamics (Xiong et al., 2017). We speculated that NBP promotes remyelination and suppresses inflammation in VD. To test this hypothesis, in the present study we investigated the effect of NBP on remyelination and inflammation in a rat model of VD and the potential roles of the AMPK/SIRT1 and STAT3/NF-κB signaling pathways.

## Materials and Methods

### Animals

Adult male Sprague–Dawley rats (*n* = 72, 200–250 g) were purchased from the animal center of Hebei Medical University (license no. SCXK[Ji] 2018-004) and housed two per cage in the laboratory animal center of Hebei General Hospital at 24°C ± 2°C and 60%–70% humidity on a 12:12-h light/dark cycle. A 1-week adaption period was allowed before the rats were used for experiments. Food and water were freely available throughout the study. Animal procedures were approved by the Ethics Committee of Hebei General Hospital (protocol no. 201929) and were carried out in accordance with the Guide for the Care and Use of Laboratory Animals of the National Institutes of Health (Bethesda, MD, USA).

### Main Chemicals

NBP (C_12_H_14_O_2_) was a gift from Shijiazhuang Pharmaceutical Company (Shijiazhuang, China). The NBP stock solution was diluted with corn oil for gavage. 5-Bromo-2′-deoxyuridine (BrdU) was purchased from Solarbio (Beijing, China). Antibodies against the following proteins were used in this study: myelin basic protein (MBP; cat. no. ab40390), SIRT1 (cat. no. ab189494) and BrdU (cat. no. ab8152; all from Abcam, Cambridge, UK); glial fibrillary acidic protein (GFAP; cat. no. 16825-1-AP), oligodendrocyte transcription factor (Olig)2 (cat. no. 13999-1-AP), NF-κB (cat. no. 10745-1-AP), tumor necrosis factor (TNF)-α (cat. no. 17590-1-AP), and β-actin (cat. no. 20536-1-AP; all from Proteintech Biotechnology, Wuhan, China); phosphorylated (p-)STAT3 (cat. no. YP0250) and p-AMPKα1/2 (cat. no. YP0575; both from ImmunoWay, Plano, TX, USA).

### Surgical Procedure

Inducing CCH through bilateral common carotid artery occlusion (2VO) is the most frequently used method for modeling VD in rats (Shibata et al., 2007). Briefly, rats were anesthetized by intraperitoneal injection of 3% pentobarbital sodium (0.2 ml/kg; Supelco, Bellefonte, PA, USA; cat. no. P-010), and bilateral common carotid arteries were exposed and isolated with a glass nerve dissector while avoiding contact with the vagus nerve. Both common carotid arteries were double-ligated with a 4–0 silk suture and a cut was made between the ligatures before the wound was stitched shut and disinfected. Body temperature was maintained at 37°C ± 0.5°C throughout the procedure with a heating lamp. Analgesia was provided through subcutaneous injection of ketoprofen (10 mg/kg dissolved in 0.2% dimethylsulfoxide; Solarbio, cat. no. IK0050) 2 h after surgery and twice a day for two consecutive days (staggered from the gavage by 2 h). Rats were housed in individual cages after the surgery and monitored until complete recovery. Sham-operated control animals were subjected to the same procedure except that ligation and dissociation of bilateral common carotid arteries were not performed.

### Groups and Drug Administration

A total of 18 rats were randomly selected as the sham group that received vehicle (i.e., corn oil) treatment. The remaining 54 rats were randomly assigned to the following three groups after 2VO surgery (*n* = 18/group): VD model (2VO surgery + vehicle treatment); NBP60 (2VO + 60 mg/kg NBP); and NBP120 (2VO + 120 mg/kg NBP). NBP doses were selected based on our previous study (Niu et al., 2019). Gavage with NBP or vehicle was performed once a day (between 10:00 and 12:00 a.m.) from the day after surgery to the day of sacrifice (2 or 4 weeks post surgery).

### BrdU Injection

To assess cell proliferation, three rats in each group were intraperitoneally injected with BrdU (50 mg/kg in 0.9% saline) once a day for 14 days starting from 24 h after the surgery.

### Morris Water Maze (MWM) Test

Spatial learning and memory were tested with the MWM (Shanghai Jiliang Software Technology Company, Shanghai, China) 6 days before sacrifice. The maze consisted of three parts: a black circular pool with a diameter of 160 cm and depth of 45 cm; a camera system tracking the rats’ movement; and software for calculating parameters such as escape latency and the amount of time spent in the target quadrant. The pool was filled with water at 23°C ± 1°C and a platform was installed 1–2 cm below the water surface. The test comprised two phases: the place navigation test for five consecutive days, and the spatial probe test on day 6. In the first phase, the rat was placed in different quadrants of the pool on each day and allowed to search for the platform, with the camera and software system recording the rat’s swimming path. If the rat failed to locate the platform within 120 s, it was guided to the platform and allowed to remain there for 20 s. In this case, the escape latency was recorded as 120 s. For the spatial probe test, the platform was taken away. The experimental animals were placed in the water diagonally across from the original platform and allowed to search for the platform for 120 s. The number of platform crossings and the amount of time spent in the target quadrant were recorded.

### Luxol Fast Blue (LFB) Staining

After anesthetization with 3% pentobarbital sodium (0.2 ml/kg), rats were transcardially perfused with 0.9% saline followed by 4% paraformaldehyde (Solarbio; cat. no. P1110). The brain was removed and immersed in 4% paraformaldehyde for 24 h. The corpus callosum was embedded in paraffin and cut into 5-μm–thick coronal sections that were deparaffinized in xylene, absolute ethanol, and 75% ethanol before incubation in LFB solution (Servicebio; cat. no. G1030) for 4 h at 60°C. The sections were placed in 0.05% lithium carbonate solution followed by 70% ethanol to reduce background staining, then mounted on slides and sealed with neutral balsam for light microscopy examination (Eclipse E100, Nikon, Tokyo, Japan). The degree of demyelination was quantified with Image-Pro Plus v.6.0 software (Media Cybernetics, Bethesda, MD, USA).

### Immunofluorescence Double Staining

Deparaffinized brain sections were immersed in EDTA (pH 8.0) and heated in the microwave for antigen retrieval. After three washes with phosphate-buffered saline (PBS; pH 7.4), an autofluorescence quencher (Servicebio; cat. no. G1221) was added to the sections, followed by incubation for 30 min in blocking solution containing bovine serum albumin (BSA; Servicebio; cat. no. G5001). The sections were incubated overnight at 4°C with rabbit primary antibodies against GFAP and Olig2 (both used at 1:100). To detect incorporated BrdU, two extra steps were performed—i.e., DNA denaturation (1 mM HCl at 37°C) for 30 min and HCl neutralization (50 mM sodium borate buffer, pH 8.5) for 20 min. The sections were incubated overnight with a mouse primary antibody against BrdU (1:100), washed three times with PBS, and incubated with Cy3-conjugated anti-rabbit (1:300, Servicebio; cat. no. GB21302) and AlexaFluor488-conjugated anti-mouse (1:400, Servicebio; cat. no. GB25301) secondary antibodies for 50 min in the dark. Finally, the sections were stained with DAPI (Servicebio; cat. no. G1012) for 10 min, washed three times, and sealed with fluorescent mounting medium (Servicebio; cat. no. G1401). Fluorescent signals were visualized with an epifluorescence microscope (Eclipse C1; Nikon) at 40× objective and signals in three areas of the corpus callosum were quantified with an imaging system (DS-U3; Nikon). Double-positive cells were manually counted in five sections from each animal with Image-Pro Plus v6.0 software by a blinded investigator.

### Immunohistochemistry

To verify the specificity of antibody labeling, we performed immunohistochemical analysis of representative samples. Briefly, sections were deparaffinized and antigen retrieval was performed by heating in citric acid buffer (pH 6.0) in the microwave oven, followed by three washes with PBS (pH 7.4). The extra steps of DNA denaturation (1 mM HCl at 37°C) for 30 min and HCl neutralization (50 mM sodium borate buffer, pH 8.5) for 20 min were performed to detect BrdU. The sections were then incubated with 3% H_2_O_2_ for 25 min at room temperature, washed three times with PBS, blocked with 3% BSA for 30 min, and incubated at 4°C with rabbit anti-GFAP (1:400) and anti-Olig2 (1:100) and mouse anti-BrdU (1:100) primary antibodies. The next day, the sections were washed three times with PBS and incubated with horseradish peroxidase (HRP)-labeled anti-rabbit (1:200; Servicebio; cat. no. GB23303) and anti-mouse (1:200; Servicebio; cat. no. GB23301) secondary antibodies for 50 min. After three washes with PBS, the sections were incubated with diaminobenzidine, washed with water, stained with hematoxylin, and washed. The samples were then dehydrated through a graded series of alcohol, cleared in xylene, mounted with neutral balsam, and examined under a light microscope.

### Hematoxylin–Eosin Staining (H&E)

To evaluate the integrity of the hippocampus, we performed H&E staining on tissue sections of the hippocampus CA1 area of rats sacrificed 4 weeks after 2VO. Two sections of the same site were selected and examined by light microscopy.

### Western Blotting

Six rats were randomly chosen for western blot analysis. After sacrifice, the white matter was removed on an ice plate and homogenized in ice-cold radioimmunoprecipitation assay buffer (Solarbio; cat. no. R0010) according to the manufacturer’s protocol, followed by centrifugation (4°C; 12,000× *g*; 10 min) to obtain total protein. For quantification of NF-κB, nucleoproteins were extracted with the Nuclear Protein Extraction Kit (Solarbio; cat. no. R0050) according to the manufacturer’s protocol. Protein concentration was measured with the BCA Protein Assay kit (Solarbio; cat. no. PC0020), and 40 μg per sample was separated by 10% sodium dodecyl sulfate-polyacrylamide electrophoresis and electro transferred to a polyvinylidene difluoride membrane (Millipore, Beijing, China) that was blocked in 5% fat-free milk for 1 h before overnight incubation at 4°C with primary antibodies against MBP (1:1,000), Olig2 (1:500), p-STAT3 (1:1,000), NF-κB (1:1,000), SIRT1 (1:500), p-AMPK (1:1,000), and TNF-α (1:1,000) as well as β-actin (1:4,000; loading control). The following day, the membrane was washed three times with Tris-buffered saline with 0.1% Tween-20 and then incubated for 45 min at 37°C with HRP-conjugated goat anti-rabbit IgG H&L (1:5,000; Abcam; cat. no. ab6721). Protein bands were visualized with an enhanced chemiluminescence kit (Wanlei Biotechnology Company; cat. no. WLA003) and quantified with Gel-Pro-Analyzer software (Media Cybernetics). The relative intensity of each band was normalized to that of β-actin.

### Quantitative Real-Time (qRT-)PCR

Total RNA was extracted from the white matter using TRIzol reagent (Tiangen Biotech, Beijing, China; cat. no. DP419) and quantified with a NanoDrop 2000 spectrophotometer (Thermo Fisher Scientific, Waltham, MA, USA). An equal amount of RNA from each group was reverse transcribed into cDNA using a cDNA synthesis kit (Tiangen Biotech; cat. no. KR118) according to the manufacturer’s instructions. qRT-PCR was performed with SuperReal PreMix Plus (Tiangen Biotech; cat. no. FP205) on an ABI 7500 PCR System (Applied Biosystems, Foster City, CA, USA), with β-actin as an internal control. The reaction contained 2× SuperReal PreMix Plus (10 μl), 50× ROX Reference Dye (0.4 μl), forward and reverse primers (0.6 μl each), cDNA derived from 0.1 μg total RNA, and RNase-free double-distilled H_2_O (6.4 μl). All reactions were run in triplicate. The reaction conditions were as follows: 95°C for 15 min, followed by 40 cycles of 95°C for 10 s and 60°C for 32 s. Relative expression levels of target genes were determined with the 2^−ΔΔCt^ method. The following forward and reverse primers were used: MBP, GCTGGCATGTCCCTGTGTCTG and CCCAATCGCAGTCCCTTGTGAG; Olig2, TTGGCTGAACGAACACTCCG and TCCAGGGATGGATGTACCCG; TNF-α, GCGTGTTCATCCGTTCTCTAC and CTTCAGCGTCTCGTGTGTTTC; SIRT1, AACCACCAAAGCGGAAAAAAAGAA and CACAGCAAGGCGAGCATAAATA; and β-actin, TGTCACCAACTGGGACGATA and GGGGTGTTGAAGGTCTCAAA.

### Statistical Analysis

**Figure 1:**
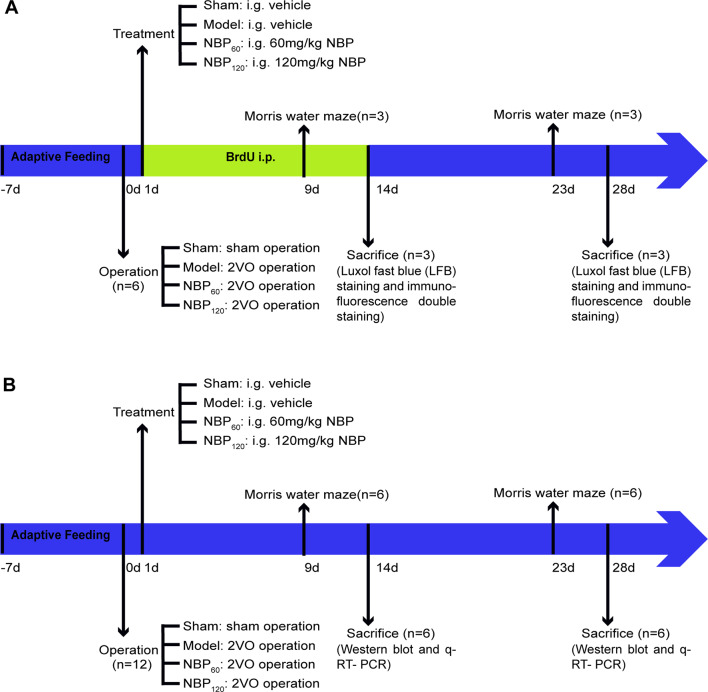
A complete flow chart of this study. **(A)** The flow chart of the morphological part in this study. The rats in each group were injected with BrdU intraperitoneally for 14 consecutive days. They were sacrificed at 2 weeks and 4 weeks separately for luxol fast blue (LFB) staining and immunofluorescence double staining. *n* = 6. **(B)** The flow chart of the molecular part in this study. Rats in each group were sacrificed at 2 weeks and 4 weeks separately for western blot and q-RT-PCR. *n* = 12.

Data are presented as mean ± standard deviation and were analyzed using SPSS v20.0 software (IBM, Armonk, NY, USA). Escape latency in the MWM was evaluated by repeated measures analysis of variance (ANOVA), and Tamhane’s T2 test was then used for multiple comparisons between groups. Intergroup comparisons at different time points (2 and 4 weeks) for the MWM spatial probe test and for LFB staining, immunofluorescence double labeling, and western blotting data (MBP, p-AMPK, NF-κB, p-STAT3, Olig2, and SIRT1 at 2 weeks and MBP, p-AMPK, NF-κB, and p-STAT3 at 4 weeks) were performed by one-way ANOVA with Fisher’s least significant difference test. Western blotting (TNF-α at 2 weeks and SIRT1, Olig2, and TNF-α at 4 weeks) and qRT-PCR data were assessed by one-way ANOVA with Tamhane’s T2 test. Data for CCH rats sacrificed at 2 and 4 weeks were compared with the independent samples *t*-test. *P* < 0.05 was considered statistically significant. A complete flow chart of the study design is shown in Figures 1A,B.

## Results

### NBP Alleviates Learning and Memory Deficits in CCH Rats

**Figure 2:**
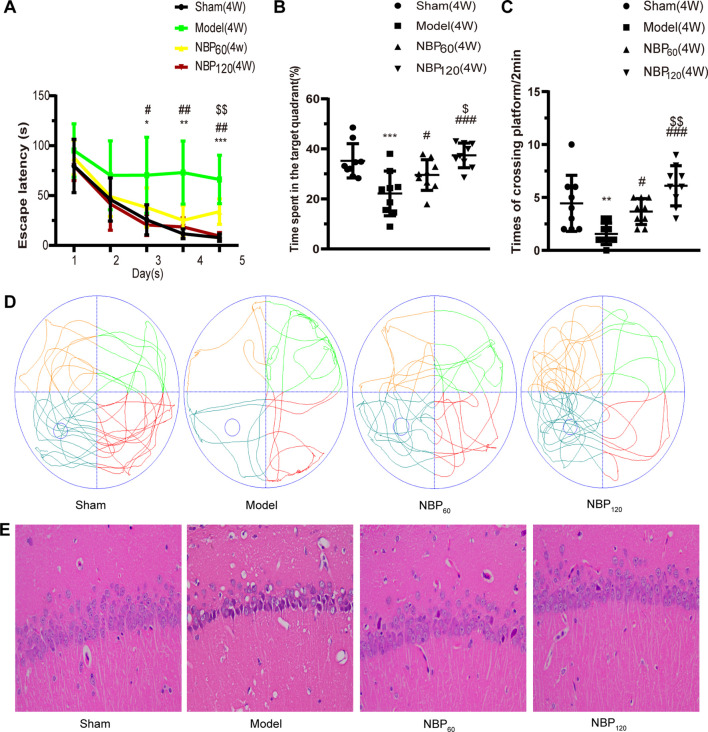
Dl-3-n-butylphthalide (NBP) alleviates learning and memory deficiency, and the pathologic changes in hippocampal CA 1 region at 4 weeks after 2VO (Morris water maze, *n* = 9 in each group; Hematox­ylin-eosin staining, *n* = 3 in each group). **(A)** Escape latency changes in different groups from day 1 to day 5. **(B)** Changes in time spent in the target quadrant (%) at day 6. **(C)** Changes in frequency of platform crossings in 2 min at day 6. **(D)** Swimming path of rats at day 6 in different groups. **(E)** Representative images of Hematoxylin-eosin staining in the hippocampal CA1 region. *p < 0.05, **p < 0.01, ***p < 0.001, the model group vs. the sham group; ^#^*p* < 0.05, ^##^*p* < 0.01, ^###^*p* < 0.001, the NBP60 group or NBP120 group vs. the model group; ^$^*p* < 0.05, ^$$^*p* < 0.01, the NBP60 group vs. NBP120 group. Values are expressed as mean ± SD.

The degree of cognitive impairment in CCH rats and the effects of NBP were investigated by testing spatial learning and memory with the MWM. At 2 weeks after 2VO, there were no significant differences in performance between groups (Supplementary Figures S1A–D) but at 4 weeks, rats in the model group showed a decline in spatial learning and memory, which was alleviated by NBP treatment. The escape latency of CCH rats was significantly longer than that of the sham group on days 3–5 (day 3: *P* < 0.05; day 4: *P* < 0.01; day 5: *P* < 0.001), while rats in the NBP-treated group had a shorter latency than untreated CCH rats (day 3: *P* < 0.05, NBP120 vs. model group; day 4: *P* < 0.05, NBP60 vs. model group and *P* < 0.01, NBP120 vs. model group; day 5: *P* < 0.05, NBP60 vs. model group and *P* < 0.01, NBP120 vs. model group). Escape latency in each group gradually decreased in the final days of training (Figure 2A). In the spatial probe test on day 6, untreated CCH rats spent less time in the target quadrant and had fewer platform crossings than those in the sham group (all *P* < 0.001). In contrast, rats in NBP-treated groups spent more time in the target quadrant and had a higher frequency of platform crossings (all *P* < 0.05, NBP60 vs. model group and *P* < 0.001, NBP120 vs. model group). Moreover, the NBP120 group spent more time in the target quadrant (*P* < 0.05) and had more platform crossings (*P* < 0.01) than the NBP60 group (Figures 2B–D).

### NBP Alleviates Pathologic Changes in the Hippocampal CA1 Region Following CCH

Neurons in hippocampus CA1 region of rats in the sham group had a distinct shape and were of moderate size, with normal microstructure. In contrast, neuronal shrinkage and loss and pyknosis of the cytoplasm was observed in 2VO rats. Administration of NBP partly reversed these morphologic changes (Figure 2E).

### NBP Reduces Demyelination in the Corpus Callosum Following CCH

**Figure 3:**
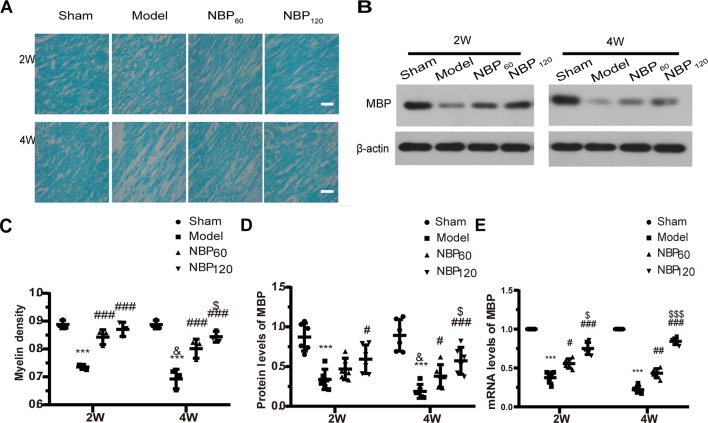
Dl-3-n-butylphthalide (NBP) attenuates demyelination in corpus callosum after 2VO. **(A)** Representative images of luxol fast blue (LFB) staining in the corpus callosum at 2 weeks and 4 weeks after 2VO. Bar = 50 μm (*n* = 3 in each group). **(B)** Western blot analysis of the expressions of MBP (*n* = 6 in each group). **(C)** Myelin density in each group presented by the percentage of stained area in total area of corpus callosum. **(D)** Quantitative analysis of protein levels of MBP. β-actin was used as an internal control. **(E)** Quantitative analysis of mRNA levels of MBP. β-actin was used as an internal control (*n* = 6 in each group). ***p < 0.001, the model group vs. the sham group; ^#^*p* < 0.05, ^##^*p* < 0.01, ^###^*p* < 0.001, the NBP_60_ group or NBP_120_ group vs. the model group; ^$^*p* < 0.05, ^$$$^*p* < 0.001, the NBP60 group vs. NBP120 group; and ^&^*p* < 0.05, the model group sacrificed at 2 weeks vs. the model group sacrificed at 4 weeks. Values are expressed as mean ± SD. MBP, myelin basic protein.

At 2 weeks after 2VO, CCH rats showed decreased myelin density in the corpus callosum by LFB staining and lower MBP protein and mRNA levels compared to the sham group (all *P* < 0.001). NBP treatment alleviated demyelination in a dose-dependent manner, as evidenced by increased LFB staining (all *P* < 0.001, NBP60 or NBP120 vs. model group), MBP mRNA level (*P* < 0.05, NBP60 vs. model group and *P* < 0.001, NBP120 vs. model group), and MBP protein level (*P* < 0.05, NBP120 vs. model group; Figures 3A–E). At 4 weeks after the surgery, the model group still showed decreased myelin density and MBP protein and mRNA levels relative to the sham group (all *P* < 0.001); moreover, demyelination was more severe in these rats compared to those sacrificed at 2 weeks (all *P* < 0.05; Figures 3A–D), reflecting the progressive loss of myelin over time after CCH. By comparison, rats in NBP-treated groups showed less demyelination by LFB staining (all *P* < 0.001, NBP60 or NBP120 vs. model group) and higher MBP protein level (*P* < 0.05, NBP60 vs. model group and *P* < 0.001, NBP120 vs. model group) and MBP mRNA level (*P* < 0.01, NBP60 vs. model group and *P* < 0.001, NBP120 vs. model group), with greater improvement observed in the NBP120 group than in the NBP60 group (LFB staining: *P* < 0.05; protein levels of MBP: *P* < 0.05; mRNA levels of MBP: *P* < 0.001), demonstrating a dose-dependent protective effect (Figures 3A–E).

### NBP Reduces Oligodendrocyte Loss and Promotes Oligodendrocyte Regeneration in the Corpus Callosum

To confirm the specificity of antibody labeling, we performed immunohistochemistry on representative coronal brain sections and found that anti-Olig2 and -GFAP antibodies labeled oligodendrocytes and astrocytes, respectively, whereas BrdU labeling was restricted to the nucleus (Supplementary Figure S2).

**Figure 4:**
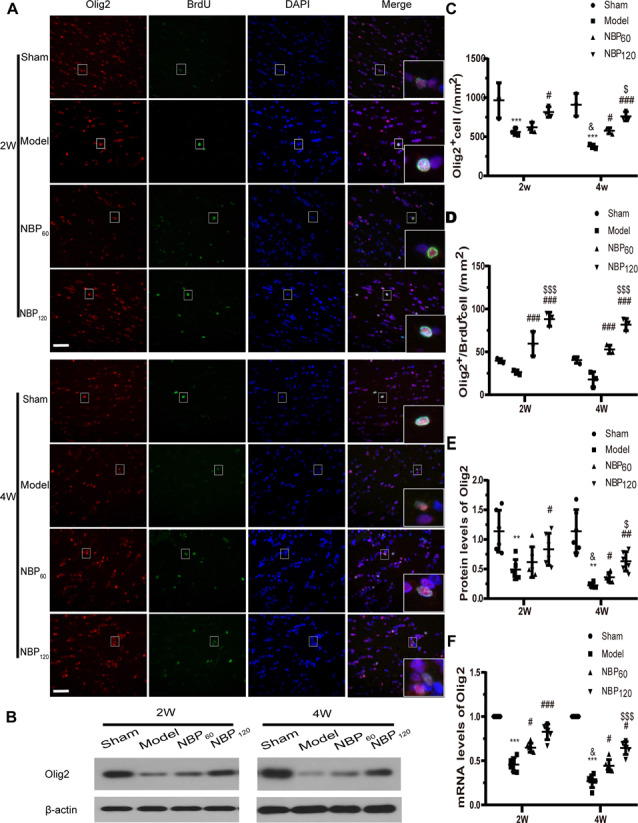
Dl-3-n-butylphthalide (NBP) promotes oligodendrocytes regeneration in corpus callosum after 2VO. **(A)** Representative images of Olig2 (red) and BrdU (green) immunofluorescence double labeling in corpus callosum at 2 weeks and 4 weeks after 2VO. Bar = 50 μm. Typical double-labeled areas are magnified (*n* = 3 in each group). **(B)** Western blot analysis of the expressions of Olig2 (*n* = 6 in each group). **(C)** The number of Olig2+ cells in corpus callosum. **(D)** The number of Olig2+/BrdU+ cells in corpus callosum. **(E)** Quantitative analysis of protein levels of Olig2. β-actin was used as an internal control. **(F)** Quantitative analysis of mRNA levels of Olig2. β-actin was used as an internal control (*n* = 6 in each group). **p < 0.01, ***p < 0.001, the model group vs. the sham group; ^#^*p* < 0.05, ^##^*p* < 0.01, ^###^*p* < 0.001, the NBP60 group or NBP120 group vs. the model group; ^$^*p* < 0.05, ^$$$^*p* < 0.001, the NBP60 group vs. NBP120 group; and ^&^*p* < 0.05, the model group sacrificed at 2 weeks vs. the model group sacrificed at 4 weeks. Values are expressed as mean ± SD. Olig2, oligodendrocyte lineage transcription factor 2.

The number of oligodendrocytes in the corpus callosum was quantified by Olig2 immunolabeling. Compared to the sham group, the number of Olig2+ cells decreased over time in untreated CCH rats (all *P* < 0.001, model group sacrificed at 2 and 4 weeks vs. sham group and *P* < 0.05, model group sacrificed at 2 weeks vs. at 4 weeks). However, the NBP120 group had more Olig2+ cells than the model group at 2 and 4 weeks (*P* < 0.05 at 2 weeks, *P* < 0.001 at 4 weeks; Figures 4A,C). We quantified the number of Olig2+/BrdU+ cells as an index of oligodendrocyte regeneration. Compared to untreated CCH rats, those treated with NBP had more Olig2+/BrdU+ cells at 2 and 4 weeks (all *P* < 0.001, NBP60 or NBP120 vs. model group; Figures 4A,D). Additionally, while Olig2 protein and mRNA levels were lower in model rats than in sham rats at both postsurgery time points (protein level of Olig2: *P* < 0.01; mRNA level of Olig2: *P* < 0.001), higher levels were observed in the NBP120 group at 2 weeks (protein level of Olig2: *P* < 0.05; mRNA level of Olig2: *P* < 0.001) and at 4 weeks (protein level of Olig2: *P* < 0.01; mRNA level of Olig2: *p* < 0.05; Figures 4B,E,F).

### NBP Suppresses Astrocyte Proliferation Following CCH

**Figure 5:**
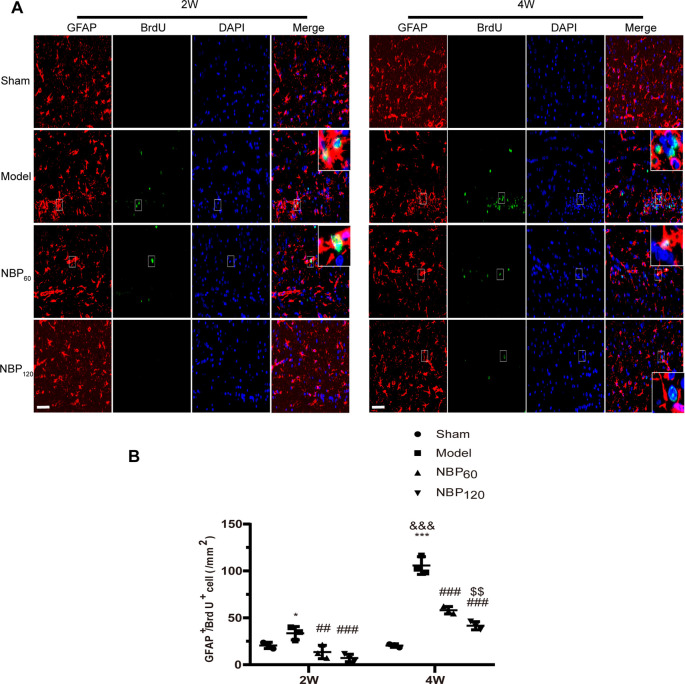
Dl-3-n-butylphthalide (NBP) suppresses astrocytes regeneration after 2VO. **(A)** Representative images of GFAP (red) and BrdU (green) immunofluorescence double labeling in corpus callosum at 2 weeks and 4 weeks after 2VO. Bar = 50 μm. Typical double-labeled areas are magnified (*n* = 3 in each group). **(B)** The number of GFAP+/BrdU+ cells in corpus callosum. *p < 0.05, ***p < 0.001, the model group vs. the sham group; ^##^*p* < 0.01, ^###^*p* < 0.001, the NBP60 group or NBP120 group vs. the model group; ^$$^*p* < 0.01, the NBP60 group vs. NBP120 group; ^&&&^*p* < 0.001, the model group sacrificed at 2 weeks vs. the model group sacrificed at 4 weeks. Values are expressed as mean ± SD. GFAP, glial fibrillary acidic protein.

We performed double immunofluorescence labeling of GFAP and BrdU to detect astrocyte regeneration. Compared to rats in the sham group, CCH rats showed an increased number of GFAP+/BrdU+ double-positive cells at 2 weeks (*P* < 0.05) and 4 weeks (*P* < 0.001). Treatment with NBP reduced astrocyte regeneration at 2 weeks (*P* < 0.01 NBP60 vs. model group; *P* < 0.001 NBP120 vs. model group) and 4 weeks (all *P* < 0.001, NBP60 or NBP120 vs. model group; Figures 5A,B).

### NBP Suppresses Inflammation Induced by CCH

**Figure 6:**
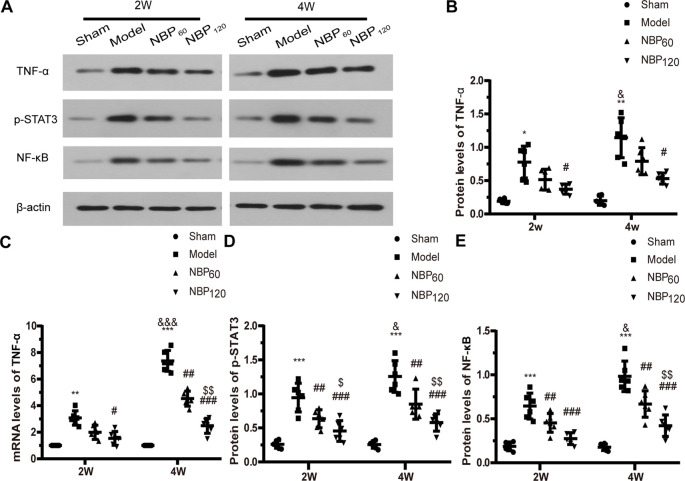
Dl-3-n-butylphthalide (NBP) suppresses inflammation induced after 2VO. **(A)** Western blot analysis of the expressions of TNF-a, p-STAT3 and NF-KB (*n* = 6 in each group). **(B)** Quantitative analysis of protein levels of TNF-α. **(C)** Quantitative analysis of mRNA levels of TNF-α (*n* = 6 in each group). **(D)** Quantitative analysis of protein levels of p-STAT3. **(E)** Quantitative analysis of protein levels of NF-KB. β-actin was used as an internal control. *p < 0.05, **p < 0.01, ***p < 0.001, the model group vs. the sham group; ^#^*p* < 0.05, ^##^*p* < 0.01, ^###^*p* < 0.001, the NBP60 group or NBP120 group vs. the model group; ^$^*p* < 0.05, ^$$^*p* < 0.01, the NBP60 group vs. NBP120 group; ^&^*p* < 0.05, ^&&&^*p* < 0.001, the model group sacrificed at 2 weeks vs. the model group sacrificed at 4 weeks. Values are expressed as mean ± SD. TNF-α, tumor necrosis factor-α. p-STAT3, phosphorylated signal transducers and activators of transcription 3. NF-κB, nuclear factor κB.

To investigate whether the protective effects of NBP against demyelination and cognitive dysfunction following CCH involve the suppression of inflammation, we measured the protein levels of TNF-α, p-STAT3, and NF-κB by western blotting and the mRNA level of TNF-α by qRT-PCR (Figures 6A–E). At 2 weeks after 2VO, CCH rats showed elevated expression of these inflammatory cytokines compared to the sham group (protein level of TNF-α: *P* < 0.05; protein level of p-STAT3: *P* < 0.001; protein level of NF-κB: *P* < 0.001; mRNA level of TNF-α: *P* < 0.01). However, treatment with NBP partly reversed this effect (protein level of TNF-α: *P* < 0.05, NBP120 vs. model group; protein level of p-STAT3: *P* < 0.001, NBP120 vs. model group and *P* < 0.01, NBP60 vs. model group; protein level of NF-κB: *P* < 0.001, NBP120 vs. model group and *P* < 0.01, NBP60 vs. model group; mRNA level of TNF-α: *P* < 0.05 NBP120 vs. model group). A similar trend was observed at 4 weeks after 2VO, with rats in the model group showing higher levels of proinflammatory cytokines than sham rats (protein level of TNF-α: *P* < 0.01; protein level of p-STAT3: *P* < 0.001; protein level of NF-κB: *P* < 0.001; mRNA level of TNF-α: *P* < 0.001). Compared to model rats sacrificed 2 weeks after 2VO in the same group, those sacrificed at 4 weeks had more severe inflammation (protein levels of TNF-α, p-STAT3, and NF-κB: all *P* < 0.05; mRNA level of TNF-α: *P* < 0.001). As expected, treatment with NBP reduced inflammation at 4 weeks (protein level of TNF-α: *P* < 0.05, NBP120 vs. model group; protein level of p-STAT3: *P* < 0.001, NBP120 vs. model group and *P* < 0.01, NBP60 vs. model group; protein level of NF-κB: *P* < 0.001, NBP120 vs. model group and *P* < 0.01, NBP60 vs. model group; mRNA level of TNF-α: *P* < 0.001, NBP120 vs. model group and *P* < 0.01, NBP60 vs. model group).

### NBP Activates AMPK/SIRT1 Signaling

**Figure 7:**
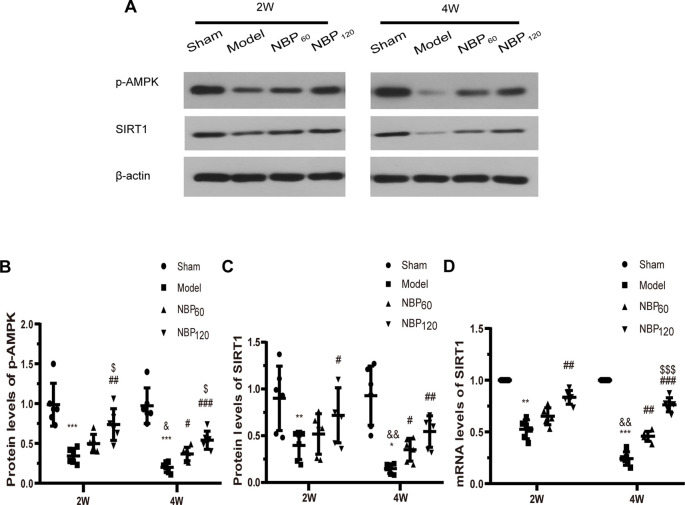
Dl-3-n-butylphthalide (NBP) upregulates AMPK/SIRT1 signaling after 2VO. **(A)** Western blot analysis of the expressions of p-AMPK and SIRT1 (*n* = 6 in each group). **(B)** Quantitative analysis of protein levels of p-AMPK. **(C)** Quantitative analysis of protein levels of SIRT1. **(D)** Quantitative analysis of mRNA levels of SIRT1 (*n* = 6 in each group). β-actin was used as an internal control. *p < 0.05, **p < 0.01, ***p < 0.001, the model group vs. the sham group; ^#^*p* < 0.05, ^##^*p* < 0.01, ^###^*p* < 0.001, the NBP60 group or NBP120 group vs. the model group; ^$^*p* < 0.05, ^$$$^*p* < 0.001, the NBP60 group vs. NBP120 group; and ^&^*p* < 0.05, ^&&^*p* < 0.01, the model group sacrificed at 2 weeks vs. the model group sacrificed at 4 weeks. Values are expressed as mean ± SD. p-AMPK, phosphorylated AMP-activated protein kinase. SIRT1, silent mating type information regulation 2 homolog 1.

To investigate whether AMPK/SIRT1 signaling is involved in the protective effects of NBP, we measured the protein levels of p-AMPK and SIRT1 by western blotting and the mRNA level of SIRT1 by qRT-PCR (Figures 7A–D). At 2 weeks after 2VO, these factors were downregulated in CCH rats relative to sham rats (protein level of p-AMPK: *P* < 0.001; protein level of SIRT1: *P* < 0.01; mRNA level of SIRT1: *P* < 0.01). However, treatment with NBP partly abrogated this decrease (protein level of p-AMPK: *P* < 0.01, NBP120 vs. model group; protein level of SIRT1: *P* < 0.05, NBP120 vs. model group; mRNA level of SIRT1, *P* < 0.01, NBP120 vs. model group). At 4 weeks after 2VO, rats in the model group had lower levels of AMPK/SIRT1 pathway components compared to the sham group (protein level of p-AMPK: *P* < 0.001; protein level of SIRT1: *P* < 0.05; mRNA level of SIRT1: *P* < 0.001). Moreover, compared to untreated CCH rats sacrificed 2 weeks after 2VO in the same group, those sacrificed at 4 weeks had decreased levels of AMPK/SIRT1 signaling factors (protein level of p-AMPK: *P* < 0.05; protein and mRNA levels of SIRT1: all *P* < 0.01). However, treatment with NBP increased their expression at 4 weeks after 2VO (protein level of p-AMPK: *P* < 0.001, NBP120 vs. model group and *P* < 0.05, NBP60 vs. model group; protein level of SIRT1: *P* < 0.01, NBP120 vs. model group and *P* < 0.05, NBP60 vs. model group; mRNA level of SIRT1: *P* < 0.001, NBP120 vs. model group and *P* < 0.01, NBP60 vs. model group).

## Discussion

This is the first study investigating the effects of NBP on remyelination and inflammation in a rat model of VD. We found that NBP alleviated pathologic changes in the hippocampus, promoted oligodendrocyte regeneration, inhibited astrocyte proliferation, and reduced demyelination and the expression of proinflammation cytokines. These effects were accompanied by a dose-dependent upregulation of AMPK/SIRT1 and downregulation of STAT3/NF-κB signaling in rats treated with NBP compared to untreated CCH rats. Our results provide evidence that NBP provides cognitive protection following CCH, likely through a mechanism involving modulation of the AMPK/SIRT1 and STAT3/NF-κB pathways. This is the first demonstration of crosstalk between AMPK/SIRT1 and STAT3/NF-κB signaling axes being implicated in VD.

Demyelination is often observed in WMLs in VD (Li et al., 2017; Hase et al., 2018). The main pathogenic mechanisms responsible for demyelination are hypoperfusion-hypoxia (Li et al., 2017) and inflammation (Saggu et al., 2016). The adult brain has very limited capacity to repair white matter (Shi et al., 2015), which explains the progressive demyelination and cognitive decline in VD. This is supported by the greater degree of myelin damage and poorer cognitive function at 4 weeks than at 2 weeks post-CCH observed in the present study. However, NBP treatment alleviated spatial learning and memory dysfunction in CCH rats, which was associated with increased hippocampal integrity, myelination, and MBP expression. Myelin loss plays an important role in cognitive decline (Li et al., 2017; Chen et al., 2020). We speculate that NBP improves cognitive function in CCH rats partly by preventing the loss and increasing the integrity of myelin. Indeed, therapeutic strategies that promote myelin repair have been linked to cognitive recovery (Hou et al., 2015; Jiang et al., 2017; Li et al., 2017). A prerequisite of remyelination is OPC proliferation and differentiation into myelinating oligodendrocytes (Franklin and Ffrench-Constant, 2008). The latter was demonstrated by the finding that demyelinating lesions in multiple sclerosis and leukoaraiosis in elderly patients contain an abundance of OPCs but lack oligodendrocytes (Wolswijk, 1998; Back et al., 2011). Adult OPCs differentiate into oligodendrocytes and astrocytes (Sun et al., 2015), which is mediated by Olig2 (Wegener et al., 2015) and STAT3 (Sun et al., 2015; Imamura et al., 2017), respectively. We found here that NBP treatment not only downregulated STAT3 and upregulated Olig2 but also induced the regeneration of Olig2-expressing cells at the expense of astrocytes. Thus, NBP administration restores myelin integrity in CCH rats at least in part by promoting oligodendrocyte regeneration and inhibiting astrocyte proliferation.

Reactive astrocytes inhibit oligodendrocyte regeneration (Back et al., 2011) and exacerbate oligodendrocyte death and demyelination (Johnstone et al., 2013; Shi et al., 2015). In the present study, CCH caused astrocyte proliferation, which was suppressed by NBP treatment. However, in addition to cell proliferation, reactive astrogliosis is characterized by hypertrophy of the cell soma, intertwining processes, and disruption of individual domains. Previous studies have shown that there are different astrocyte subtypes with distinct morphology, degrees of activity, and migratory capacity that have been implicated in brain damage (Wagner et al., 2013). In a rat model of stroke, hyperactivated astrocytes were found to have a larger volume and higher degree of branching than those exhibiting mild or moderate responses, whereas astrocytes expressing glutamine synthetase were more often located in remote areas compared to other subtypes (Wagner et al., 2013). However, we did not examine different astrocyte subtypes, which is a limitation of the present study.

We observed that inflammation contributed to pathologic white matter changes, in accordance with previous reports (Iadecola, 2013; Saggu et al., 2016). Reduced cerebral blood flow, oxidative stress, and extravasation of plasma proteins caused by CCH are all thought to induce inflammation in VD (Iadecola, 2013). Although the specific inflammatory mechanism responsible for white matter changes in CCH has not been elucidated, several factors may play a role. STAT3/NF-κB is well known to be involved in pro-inflammatory signaling (Won et al., 2013). STAT3 promotes astrocyte differentiation and inhibits oligodendrocyte fate specification (He et al., 2005; Sun et al., 2015; Imamura et al., 2017). Pharmacologic suppression or conditional deletion of STAT3 was shown to promote oligodendrocyte maturation and remyelination (Cao et al., 2010; Imamura et al., 2017; Elbaz et al., 2018). Crosstalk between STAT3 and NF-κB has been reported (Grivennikov and Karin, 2010), and STAT3-mediated NF-κB activation plays an important role in the pathogenesis of neurodegenerative diseases (Grivennikov and Karin, 2010). NF-κB activation is involved in oligodendrocyte death, demyelination, axonal loss, astrocyte inflammatory cascades, and loss of white matter integrity, which are related to CCH-induced cognitive decline (Saggu et al., 2016). Activated NF-κB positively regulates the expression of TNF-α (Neumann et al., 1999), which can also induce oligodendrocyte death and demyelination (Jurewicz et al., 2005; Caminero et al., 2011). Pharmacologic or transgenic suppression of these proinflammatory cytokines was shown to have a protective effect on oligodendrocytes and myelin (Wakita et al., 2003; Saggu et al., 2016). Consistent with the above findings, the CCH rats in our study showed elevated levels of these cytokines, which was accompanied by oligodendrocytes and myelin loss. However, treatment with NBP reduced the expression of proinflammatory factors while preserving oligodendrocytes and myelin. Thus, the protective effect of NBP on myelination is attributable to the suppression of proinflammatory signaling.

Another important hallmark of inflammation in ischemic injury is the migration of circulating immune cells to the brain driven by the activation of brain endothelial cells and chemokine secretion (Iadecola and Anrather, 2011; Möller et al., 2014; Pösel et al., 2016). Immune cell infiltration contributes to either ischemic damage or the resolution of inflammation and subsequent tissue repair in ischemic stroke, depending on different subpopulations of leukocytes and different phases of injury (Iadecola and Anrather, 2011). As such, immune cell invasion warrants more detailed investigation in the context of VD.

Previous studies have demonstrated the crosstalk between inflammation and AMPK signaling (Lyons and Roche, 2018). Inflammatory stimuli reduce AMPK phosphorylation in nutrient-rich conditions (Yang et al., 2010). As a downstream target of AMPK, SIRT1 can be suppressed by oxidative stress in addition to inflammation (Ota et al., 2012). These findings may explain the downregulation of AMPK/SIRT1 signaling in 2VO, which is characterized by inflammation and oxidative stress. However, activated AMPK upregulates the level of nicotinamide adenosine dinucleotide to enhance SIRT1 activity and subsequent deacetylation of the RelA/p65 subunit, resulting in inhibition of NF-κB nuclear entry and subsequent inflammation (Ashburner et al., 2001; Yeung et al., 2004; Lee et al., 2009; Salminen et al., 2011; Kauppinen et al., 2013). The regulatory mechanism responsible for the crosstalk between inflammation and AMPK/SIRT1 signaling has not been elucidated. However, in the present study, low levels of nuclear NF-κB and its downstream target TNF-α were associated with the upregulation of the AMPK/SIRT1 pathway in NBP-treated rats. Thus, NBP may function as a regulator between inflammation and AMPK/SIRT1 signaling. Given the crucial impact of inflammation on oligodendrocyte death and demyelination and the finding that AMPK/SIRT1 signaling promotes remyelination and neuroprotection (Elbaz et al., 2018; Dembic et al., 2019; Houshmand et al., 2019), we speculate that the molecular basis for the neuroprotective effect of NBP involves the upregulation of the AMPK/SIRT1 and downregulation of the STAT3/NF-κB pathway. We also observed the opposite trend for these two pathways, suggesting that they cross regulate in VD.

This study has several limitations. First, the sample sizes were small, particularly for the histologic analysis. Second, we examined only two post surgery time points because previous studies and our preliminary experiments have shown that significant pathologic changes are observed at 2 and 4 weeks after CCH (Xiong et al., 2017; Li et al., 2019b). In future studies, we will include more time windows that allow assessment of cognitive improvement. Third, we only examined GFAP+/BrdU+ cells in our quantitative analysis of astrocytes. However, given the variety of morphologies and subtypes, astrocytes warrant more detailed investigation in the context of VD. Finally, we used 2VO to mimic inflammation and white matter changes in VD. However, VD comprises many types of vascular disease including large vessel disease with multiple strokes, small vessel disease (SVD) with lacunar infarcts, and white matter disease; there are no established models that recapitulate all of the pathologic changes in VD. Spontaneously hypertensive rats (SHRs) exhibit WMLs, blood brain barrier leakage, and immune activation induced by long-term vascular changes (Kaiser et al., 2014); it would therefore be of interest to evaluate the protective effect of NBP in the SHR model.

In conclusion, the findings of this study suggest that NBP may be a promising treatment for alleviating cognitive dysfunction and promoting remyelination in VD, and that the AMPK/SIRT1 and STAT3/NF-κB pathways serve as potential targets for future therapies.

## Data Availability Statement

The dataset supporting the conclusions of this article will be made available by the authors, without undue reservation, to any qualified researcher.

## Ethics Statement

The animal study was reviewed and approved by the Ethics Committee of Hebei General Hospital.

## Author Contributions

ML and PL conceived and designed the experiments. ML, NM, XG, ZZ, WW, and XX performed the experiments. ML and XN analyzed the data. ML wrote the manuscript. PL revised the manuscript. All authors mentioned in the article have significantly contributed to the research, and read and approved the final manuscript.

## Conflict of Interest

The authors declare that the research was conducted in the absence of any commercial or financial relationships that could be construed as a potential conflict of interest.
