# Supplementary figures and images for: Dl-3-n-Butylphthalide Promotes Remyelination and Suppresses Inflammation by Regulating AMPK/SIRT1 and STAT3/NF-κB Signaling in Chronic Cerebral Hypoperfusion
Source: Front Aging Neurosci. 2020 Jun 9;12:137. doi: 10.3389/fnagi.2020.00137 (PMC7296049; doi:10.3389/fnagi.2020.00137)

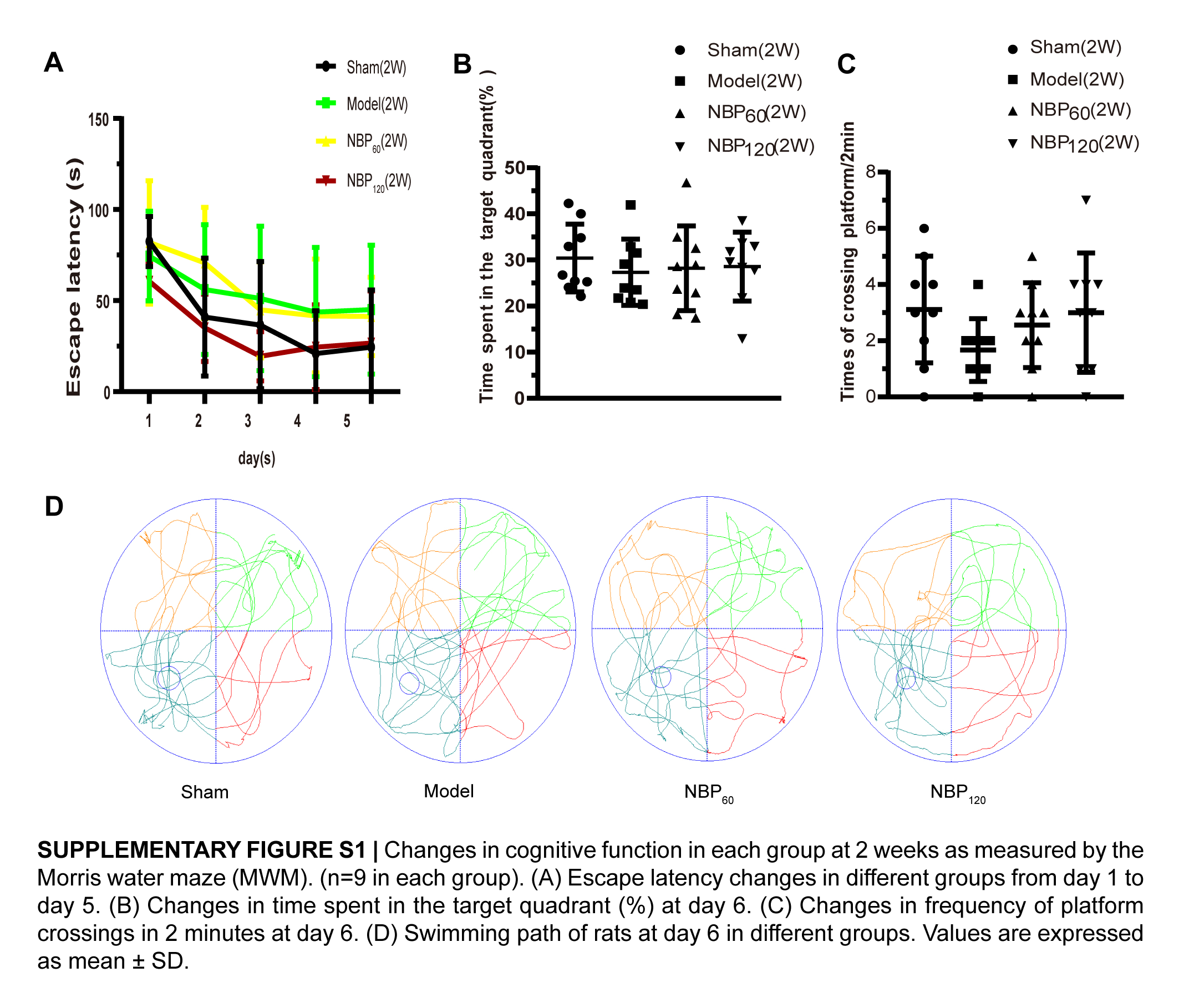

Supplement: Supplementary file 1 [file Image_1.TIF]

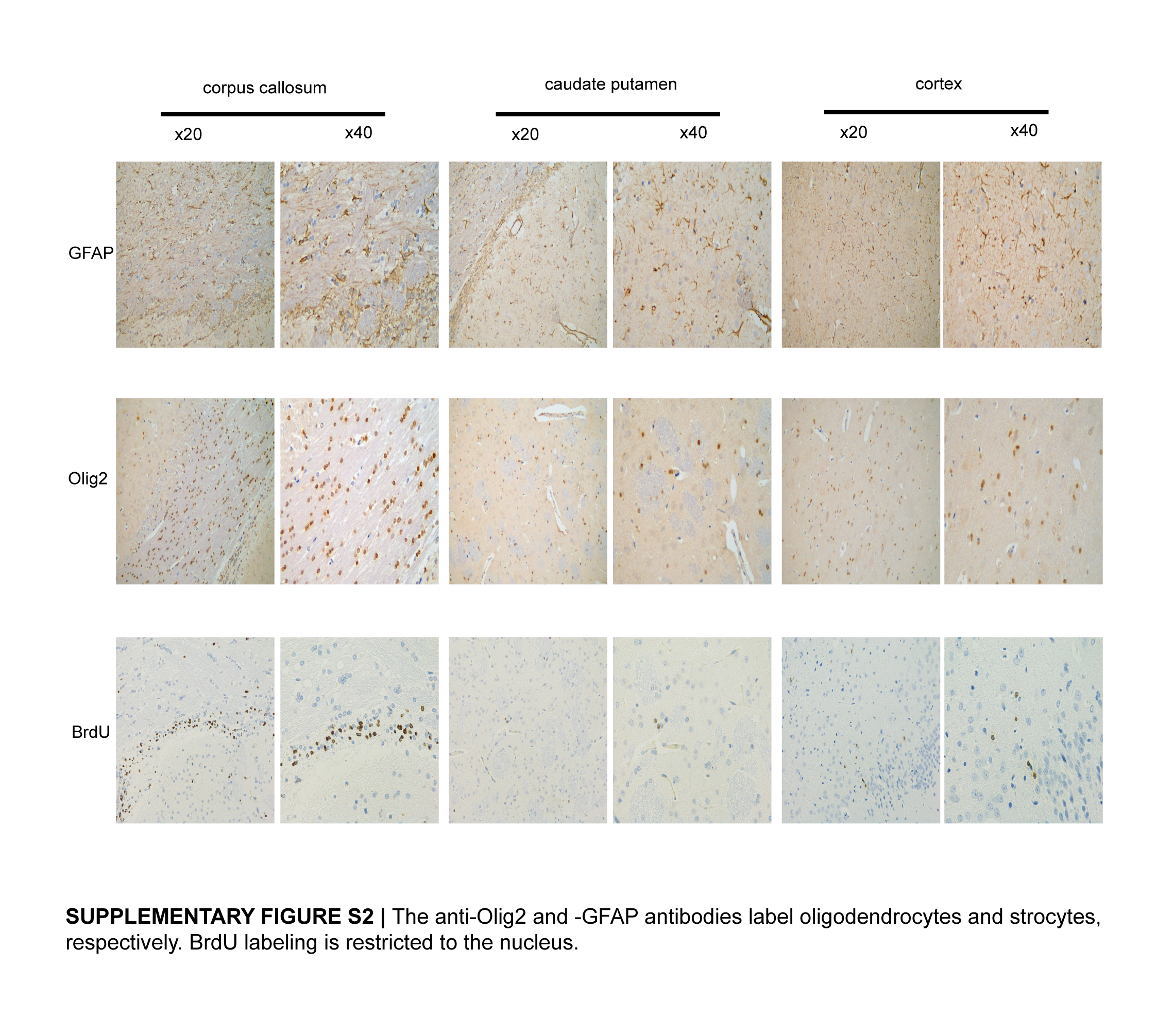

Supplement: Supplementary file 2 [file Image_2.TIF]
